# Metastatic Deposits of Breast Lobular Carcinoma to Small Bowel and Rectum

**DOI:** 10.4061/2011/413949

**Published:** 2011-07-26

**Authors:** W. Eljabu, G. Finch, J. Nottingham, N. Vaingankar

**Affiliations:** Departments of Plastic and Reconstructive Surgery, General Surgery, and Histopathology, Northampton General Hospital, Northampton NN1 5BD, UK

## Abstract

Breast cancer is the most frequent malignancy in women accounting for approximately 32% of all cancers, with a lifetime risk of 1 in 10. It causes considerable morbidity and mortality. Recently, the survival rate has dramatically increased due to early detection of the disease and improvement in the treatment measures. However, more than 30% of the patients develop metastatic diseases following surgical treatment, radiotherapy, hormonal therapy, or chemotherapy. Distant spread is usually found in bones, lungs, liver, brain and skin. Rarely, it spreads to bowel, spleen, gallbladder, pancreas, urinary bladder, and eyes. Breast cancer is the second commonest primary tumour responsible for gastrointestinal metastases after malignant melanoma. We report a case of a Caucasian female who developed an intestinal obstruction secondary to metastatic deposits to the small bowel and later to the rectum from breast lobular carcinoma 2 years after mastectomy, axillary clearance, radiotherapy, hormonal therapy, and transverse rectus abdominis myocutaneous (TRAM) flap for reconstruction.

## 1. Case Presentation

In September 2008, a 70 years old female was diagnosed with a lobular carcinoma after a core biopsy of a mass in her left breast. A month later, she underwent skin sparing mastectomy and axillary clearance, the histological examination confirmed a grade 2 invasive lobular carcinoma, estrogen receptor positive (ER+), progesterone receptor negative (PR−), and human epidermal growth factor receptor 2 negative (Her2−). There was no vascular space invasion, no in situ component and the axillary tissue included eight lymph nodes, three of those contained metastatic lobular carcinoma with extracapsular spread. A CT scan to chest and abdomen and a bone scan showed no evidence of any metastatic disease. Subsequently, she had radiotherapy and was started on adjuvant hormonal treatment, but because of her past medical history which includes previous nephrectomy for pelvic ureteric junction (PUJ) obstruction 10 year ago, arthritis and essential hypertension, chemotherapy was not considered. In early 2010, the patient was referred to the Department of Plastic Surgery for breast reconstruction, as she continued to show no clinical evidence of any recurrence, she underwent reconstruction with a pedicle transverse rectus abdominis myocutaneous (TRAM) flap.

**Figure 1:**
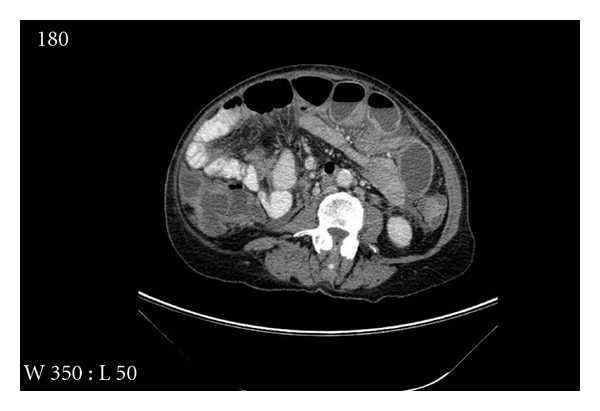
CT scan shows small bowel dilatation, no other specific features.

One month later she had multiple episodes of abdominal pain and constipation which needed hospital admission. A CT scan showed evidence of small bowel obstruction (Figure 1); a laparotomy and resection of a thickened and narrowed terminal ileum were carried out followed by bowel anastomosis. The histological analysis confirmed the presence of widely infiltrated small bowel by well-differentiated epithelial cells, some of which contained intracytoplasmic lumina and formed a single file pattern of infiltration (Figure 4). The appearances were those of metastatic lobular carcinoma of breast, which was ER positive and E-cadherin & Her 2 negative (Figure 5), invaded the mucosa, the mesentery, and appeared to extend beyond the mesenteric margins of excision. A subsequent chest/abdomen CT scan and a bone scan showed no signs of other metastasis. Contralateral carcinoma was excluded by clinical examination and mammogram. The patient was started on a new hormone therapy.

**Figure 2:**
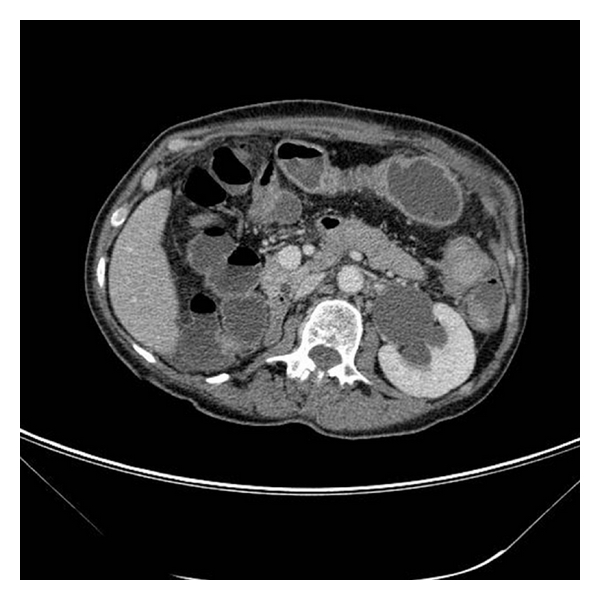
CT scan reveals left hydronephrosis secondary to compression on lower ureter by a rectal mass, previous right nephrectomy.

**Figure 3:**
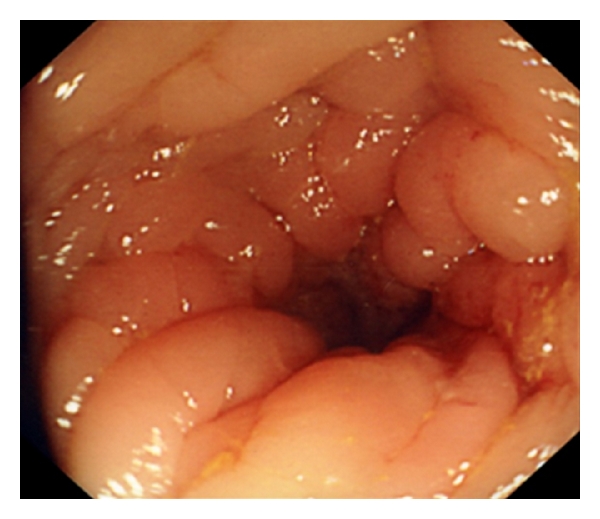
View at sigmoidoscopy showing narrowed lumen, which was felt to be due to a firm and swollen mucosa by the endoscopist.

**Figure 4:**
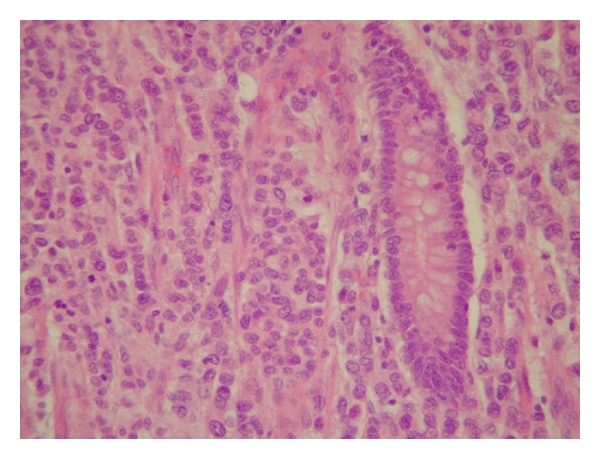
Small bowel mucosa infiltrated by cords of metastatic lobular adenocarcinoma cells. (Haematoxylin and eosin, original magnification ×400.)

**Figure 5:**
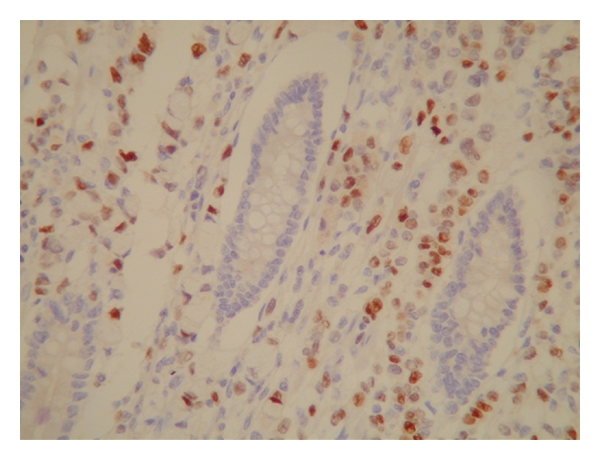
The same slide stained with antibody to estrogen receptor. The malignant cells stain brown (ER clone 6F11, original magnification ×400).

Unfortunately, 3 months later, the patient had new episodes of diarrhoea with fresh blood; sigmoidoscopy showed a narrowed lumen and firm swollen mucosa at the upper rectum (Figure 3); biopsies revealed the presence of metastatic lobular carcinoma of breast, the tumour foci stained positive with cytokeratin 7 (CK7), pancytokeratins, and ER. Staging CT scan identified left hydronephrosis caused by compression on the ureter from the metastasis in the rectum (Figure 2). No extraintestinal spread reported; the patient had a ureteric stent put in, and she was subsequently started on palliative therapy.

## 2. Discussion

The most common metastatic sites from breast cancer are well known, the lungs, bones, liver, and brain whereas the involvement of the gastrointestinal tract remains very unusual. Borst published a large series that analyzed more than 2500 cases of breast cancer with metastatic disease over a period of 18 years; he found that only 17 patients (less than 1%) had metastasis to the GI tract [1]. Metastasis to the stomach and small bowel have been more frequently reported than colonic and rectal ones. However, in a retrospective study, McLemore reported 23 cases of GI metastasis, he found that the disease is more frequent in the mid- and hindgut than in the foregut (oesophagus 8%, stomach 28%, small intestine 19% and colon-rectum 45%) [2]. Despite the greater prevalence of infiltrative ductal breast carcinoma among women (90%), lobular breast cancer has a specific metastatic pattern and more frequently metastasizes to the GI tract and the retroperitoneal tissue than ductal cancer [3]. McLemore also reported that infiltrating lobular carcinoma represented 64% of the gastrointestinal metastasis. It has also been reported that even in patients with a mixed ductal and lobular primary breast carcinoma, the lobular type is the most likely to cause metastatic disease [4].

The presenting symptoms and signs of metastatic disease to the GI tract are usually nonspecific, often mimicking other gastrointestinal disorders; this patient presented with multiple episodes of intermitted abdominal pain and restrain of stool; the initial thought was that she might had adhesional obstruction or incarcerated incisional hernia as a complication of the pedicel TRAM, but clinically there was no evidence of hernias. Diagnosis of GI metastasis from breast cancer could be difficult prior to surgical intervention; the findings of diagnostic imaging are frequently not specific for GI metastasis; the picture can also mimic Crohn's disease [5]. GI endoscopy with deep biopsy is widely used for accurate diagnosis depending on the patient status [6].

Santini et al. reported an interesting case where an increase in cancer antigen CA 19.9 was used to diagnose metastatic lobular breast cancer to ileocecal valve in a symptomatic patient [7]. Establishing the histological origin could be challenging, metastases of lobular carcinomas have a typical fashion of spread with intramural infiltration growing within the serosal, muscular, and submucosal layers with small cells in cords throughout the tissue [8]. Compared to primary adenocarcinoma of bowel, the metastatic lobular carcinoma is usually positive for CK 7, ER, PR and GCDFP 15 and negative for vimentin, while CK 20 and CEA are almost invariably present in primary gastrointestinal tumours and absent in breast carcinomas [9, 10]. In this case, the metastatic disease was positive for ER, and negative for PR and HER2, which matched the immunohistochemical findings of the primary breast cancer; this finding, along with the unique histological pattern of the disease, favoured the diagnosis of metastatic lobular breast carcinoma. 

The management of metastatic breast cancer to GI tract is still controversial. It may involve systemic hormonal or chemotherapy either alone or combined with surgery since patients usually present with the involvement of multiple organs, despite not detecting extensive disease in preoperative investigation [11]. Patients with intestinal obstruction, perforation, or bleeding require palliative surgical intervention but that does not extend the patient survival. Interestingly, Tang et al. reported 2 cases when the intestinal obstruction due to metastatic lobular carcinoma was settled with a parenteral endocrine hormonal agent (fulvestrant) [12]. Long-term survival rate has been reported after resection of an isolated GI metastatic lesion whilst surgery combined with chemotherapy may result in long-term remission [7, 13]. Bowel surgery in postmastectomy patients who have undergone Transverse Rectus Abdominis Myocutaneous (TRAM) flap would require careful preoperative planning of surgical incision, and the need for stoma cannot be ruled out [14, 15].

In summary, metastasis to GI tract from breast cancer is a rare condition and not easy to diagnose. This diagnosis should be suspected when the patient has a history of breast cancer especially of lobular type. Not surprisingly, in most cases, GI metastasis is part of widespread disease, and early diagnosis may help towards the improvement of the outcome. This case also emphasizes the importance of patients' suitability for breast reconstruction, despite the patient herself making the decision to go for TRAM flap as a means of delayed reconstruction after knowing the pros and cons, less invasive procedures and techniques should be made available and must be considered in patients with a kind of cancer that carries high risks of recurrence.
